# The benefits of permutation-based genome-wide association studies

**DOI:** 10.1093/jxb/erae280

**Published:** 2024-07-02

**Authors:** Maura John, Arthur Korte, Dominik G Grimm

**Affiliations:** Technical University of Munich, Campus Straubing for Biotechnology and Sustainability, Bioinformatics, Petersgasse 18, 94315 Straubing, Germany; Weihenstephan-Triesdorf University of Applied Sciences, Bioinformatics, Petersgasse 18, 94315 Straubing, Germany; University of Würzburg, Faculty of Biology, Julius-von-Sachs Institute, Julius-von-Sachs-Platz 3, 97082 Würzburg, Germany; Technical University of Munich, Campus Straubing for Biotechnology and Sustainability, Bioinformatics, Petersgasse 18, 94315 Straubing, Germany; Weihenstephan-Triesdorf University of Applied Sciences, Bioinformatics, Petersgasse 18, 94315 Straubing, Germany; Technical University of Munich, TUM School of Computation, Information and Technology, Boltzmannstraße 3, 85748 Garching, Germany; University of South Bohemia in České Budějovice, Czech Republic

**Keywords:** Arabidopsis, genome-wide association studies (GWAS), GPU, linear mixed models, multiple hypothesis testing, permutations

## Abstract

Linear mixed models (LMMs) are a commonly used method for genome-wide association studies (GWAS) that aim to detect associations between genetic markers and phenotypic measurements in a population of individuals while accounting for population structure and cryptic relatedness. In a standard GWAS, hundreds of thousands to millions of statistical tests are performed, requiring control for multiple hypothesis testing. Typically, static corrections that penalize the number of tests performed are used to control for the family-wise error rate, which is the probability of making at least one false positive. However, it has been shown that in practice this threshold is too conservative for normally distributed phenotypes and not stringent enough for non-normally distributed phenotypes. Therefore, permutation-based LMM approaches have recently been proposed to provide a more realistic threshold that takes phenotypic distributions into account. In this work, we discuss the advantages of permutation-based GWAS approaches, including new simulations and results from a re-analysis of all publicly available Arabidopsis phenotypes from the AraPheno database.

## Teaser

Permutation-based linear mixed model strategies for GWAS provide more realistic significance thresholds for phenotypic distributions, improving detection accuracy over static corrections, as shown in a re-analysis of >500 Arabidopsis phenotypes.

## Introduction

Genome-wide association studies (GWAS) represent a powerful and widely employed tool for investigating the relationship between genetic variations and phenotypic differences across a population of individuals. The goal of GWAS is usually to gain information about the genetic architecture of traits or to identify genetic markers that are associated with specific phenotypes or diseases (Gumpinger *et al*., 2018). One of the key benefits of GWAS lies in its ability to identify candidate genes and markers associated with traits of interest without *a priori* knowledge of the underlying biological mechanisms. By analysing the genetic variations present in Arabidopsis or other species, researchers have pinpointed novel loci that contribute significantly to phenotypic variation (Atwell *et al*., 2010; Zhao *et al*., 2011; Li *et al*., 2014; Lin *et al*., 2014; Liu *et al*., 2015; Satbhai *et al*., 2017; Falcke *et al*., 2018; Yuan *et al*., 2020; Yong *et al*., 2021). This information helps to decipher the genetic architecture of traits, and sheds light on the molecular pathways and regulatory networks involved (John *et al*., 2023a). The gold standard would be to demonstrate a causal relationship between associated variants and the trait of interest. Although this is a desirable goal—and of paramount importance for expanding our knowledge of the genotype–phenotype map or for finding candidate genes for crop improvement (Bararyenya *et al*., 2020; Thapa *et al*., 2020)—for many applications, the correlation of a genomic region with a trait may be sufficient. For example, in plant or animal breeding, correlation may be sufficient to estimate breeding values to select the most promising genotypes (Macciotta *et al*., 2009). However, whether we are interested in causality or correlation, pursuing specific candidate genes, or making claims about genetic architecture, reliable significance thresholds are imperative to distinguish true from spurious associations.

GWAS involve the simultaneous assessment of genetic variants across the entire genome to identify associations with phenotypic traits through statistical hypothesis tests. Here for each genetic marker, a so called test statistic and a corresponding *P*-value are computed. This *P*-value tells us how likely it is to receive a test statistic as extreme as the observed one under the assumption of no association with the given trait. The smaller this *P*-value the less likely it is to get such a test statistic if the marker is not associated with the phenotype. If the *P*-value is small enough, we can discard our initial assumption, that is, reject the null hypothesis, and consider the tested marker as significantly associated. For this purpose, we need an appropriate significance threshold to decide when a *P*-value is small enough. Establishing significance thresholds is a crucial step in GWAS to differentiate between true associations and random fluctuations that can be due to the massive amount of tests performed as well as through model misspecifications. Typically, the significance threshold is set based on the desired genome-wide significance level, often referred to as α. This significance level represents the probability of making a false positive association, that is, considering a marker to be significantly associated when there is no association. In standard statistics, a value of α=0.05 is often considered. Now, since we test millions of genetic variants in a typical GWAS, we need to correct for this massive amount of tests. For example, if we assume a significance level of α=5% and test 1000 markers, we expect to get 50 significant associations by chance alone; with a million markers, the number of random associations rises to 50 000.

A common approach to account for the burden of multiple testing is to control the family-wise error rate (FWER); i.e. the probability of making at least one false positive or type 1 error. One way to approximate the FWER is the commonly used Bonferroni correction (Bonferroni, 1936; Bland and Altman, 1995). Here one computes an adjusted significance threshold by dividing the significance level α by the number of tests performed, that is, the number of single nucleotide polymorphisms (SNPs) tested. As an example, for a GWAS where one million markers are tested with α=5%, the adjusted Bonferroni significance threshold would be 0.05/10^6^=5 × 10^−8^. However, on the one hand, this simple approach is often considered too conservative with an increased risk of false negatives for normally distributed phenotypes, as many genetic markers that are tested are not completely independent, since they may be—at least partially—in linkage (Eichstaedt *et al*., 2013; Llinares-López *et al*., 2015; Grimm *et al*., 2017). On the other hand, Bonferroni correction was shown to be not stringent enough for skewed distributions leading to many false positives (John *et al*., 2022, 2023b, Preprint). In addition, a more stringent threshold may also be needed for low-frequency variants (Fadista *et al*., 2016).

As an alternative, the false discovery rate (FDR) controls the proportion of false positives among significant results. Methods such as the Benjamini–Hochberg procedure lead to a more lenient threshold, allowing for the identification of more associations while still controlling the overall FDR (Benjamini and Hochberg, 1995). While the FDR is a widely used approach in GWAS, it is not without its challenges and potential issues. The Benjamini–Hochberg procedure assumes independence of the test statistics, meaning that the significance of one variant is not influenced by the significance of another. In reality, genetic variants are often correlated due to linkage disequilibrium or population structure as mentioned before. Violation of this independence assumption can result in inaccurate FDR estimates, when using the Benjamini–Hochberg procedure (Brzyski *et al*., 2017).

In general, violation of model assumptions is a common problem for GWAS. Typical presumptions include the aforementioned independence and Gaussian distribution of the residuals, and homoscedasticity (i.e. constant variance of the residuals), which are rarely met in real biological data (Poole and O’Farrell, 1971). To overcome these limitations, transformations of the phenotypic data have been proposed. The widely used Box–Cox transformation is a power transformation used to stabilize the variance and make the data more closely approximate a normal distribution (Sun *et al*., 2013). While the Box–Cox transformation is particularly useful when dealing with data that violate assumptions of normality and homoscedasticity, there are criticisms and limitations associated with its application (Shen and Rönnegård, 2013). One concern is the interpretability of the data since GWAS are not performed with the actual phenotypic data but with a complex transformation. This also complicates the validation of potential candidate genes. It is worth noting that when comparing different traits, the power parameter λ used for the transformation is specific to each phenotype. Therefore, trait correlations will be shifted, which impedes the comparison of different results. Apart from these major concerns, the Box–Cox transformation can be sensitive to outliers and still relies on a constant variance across all levels of the independent variables. If this assumption is severely violated, the transformation may not effectively deal with heteroscedasticity (Atkinson *et al*., 2021). Finally, the transformation process inherently involves a loss of information. While the goal is to improve the normality of the data, there is a trade-off between achieving normality and preserving meaningful biological information.

**Fig. 1.**
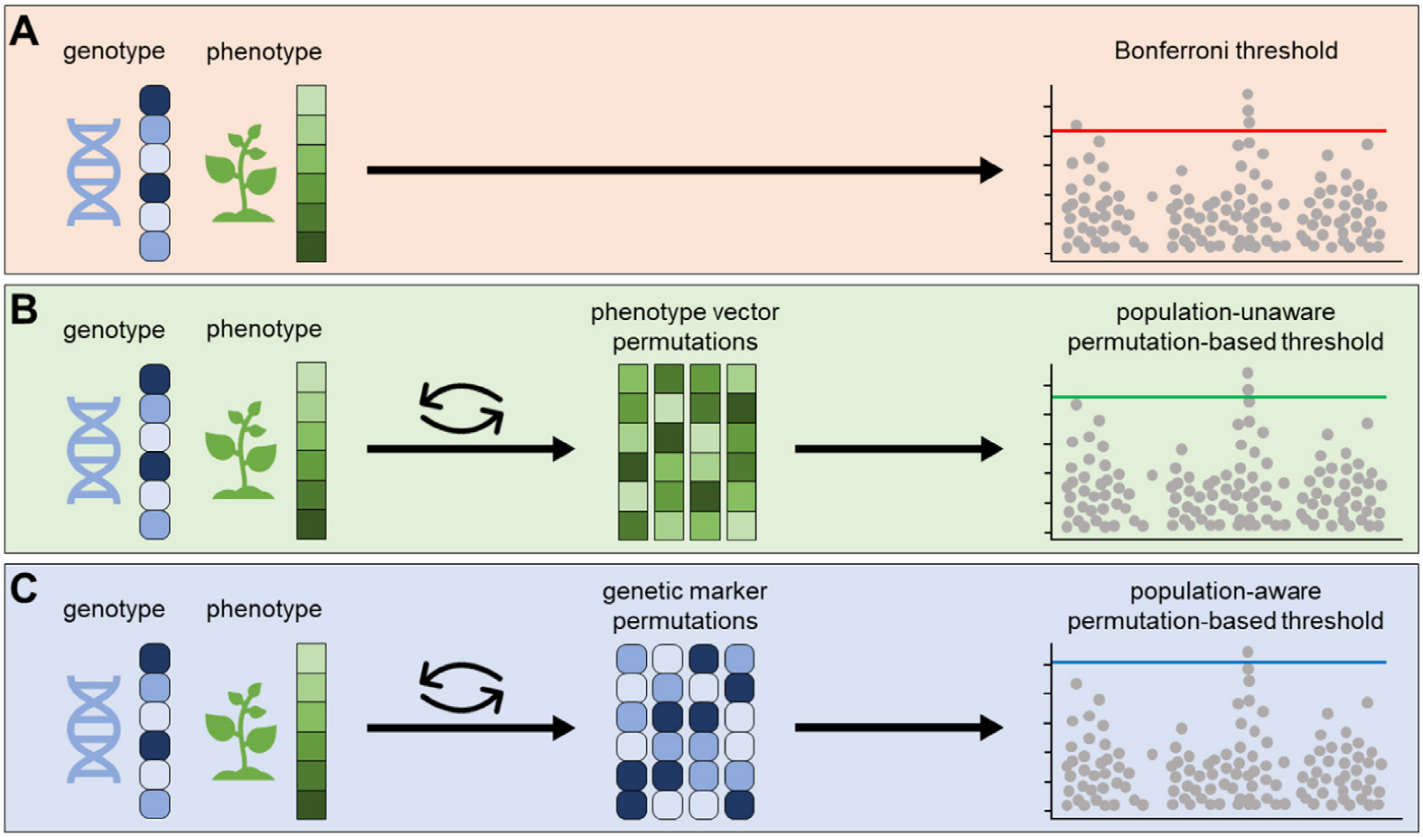
Overview of genome-wide association studies (GWAS) with permutation-based significance thresholds. (A) GWAS with classical Bonferroni significance threshold. (B) GWAS with permutation-based significance threshold, ignoring population structure by permuting the phenotype vector. (C) GWAS with permutation-based significance threshold, taking into account the population structure by permuting the genetic markers, which is equivalent to permuting the phenotype and covariance matrix.

An alternative approach to overcome some of these limitations is given by permutation-based methods (Che *et al*., 2014; Nicod *et al*., 2016; Ruzicka *et al*., 2019; Scott *et al*., 2021; John *et al*., 2022, 2023b, Preprint). Here one tries to empirically estimate the FWER by sampling the true null distribution of the test statistics. In permutation testing one can compute either adjusted *P*-values or an adjusted permutation-based threshold. However, to achieve small enough adjusted *P*-values, millions of permutations are required (Che *et al*., 2014). Therefore, John *et al*. (2022) proposed to compute an adjusted permutation-based threshold based on the *maxT* method (Westfall and Young, 1993), which is able to control the FWER with only a few hundred permutations. When computing a permutation-based significance threshold, either the phenotype values or the genetic markers are shuffled to approximate the true null distribution of the test statistics (John *et al*., 2023b, Preprint). Based on these test statistics one can derive an adjusted significance threshold for a given significance level α (Nicod *et al*., 2016; John *et al*., 2022). Figure 1 compares the workflow of a standard GWAS using a classical Bonferroni threshold with the workflow using a permutation-based threshold.

Permutation-based thresholds provide a more realistic estimate of false and true positives for GWAS and provide a different threshold for each phenotype, depending on the phenotype distribution. The main obstacle to implementing permutations used to be computational limitations, as naïvely the time to run 100 permutations would be 100 times as long as a standard GWAS. However, due to computational advances and a clever batch-wise implementation using 4D-tensors, this is no longer a major problem (John *et al*., 2022, 2023b, Preprint).

The following section describes the method and all the underlying considerations, such as the number of permutations and how to permute correctly *en detail*. Finally, we will re-analyse simulated and real phenotypes and show the benefit of permutation-based significance thresholds over classical methods.

## Materials and methods

### Permutation-based GWAS

We first review the linear mixed model framework for conducting GWAS with population structure correction. We then summarize how to mitigate common problems with multiple hypothesis correction using permutation-based techniques by discussing two alternative permutation strategies. Finally, we review some recent techniques for efficient computation of permutations.

#### Linear mixed models

Let *n* be the number of individuals and let $y\in R^{n}$ be a vector of observed phenotypic values. To determine whether a genetic marker is significantly associated with the phenotype y, we perform a statistical hypothesis test. For this, we can use a linear model, where we assume that the phenotype can be modeled as a linear combination of the genotypic values of the SNP of interest and potentially some covariates, each of them with different effect sizes (Gumpinger *et al*., 2018). A common problem with linear models, such as linear regression and logistic regression, is that results often lead to inflated test statistics due to confounding factors such as cryptic relatedness and population structure (Gumpinger *et al*., 2018). To account for these types of confounders, linear mixed models (LMMs) such as EMMA (Kang *et al*., 2008), EMMAX (Kang *et al*., 2010), or FaST-LMM (Lippert *et al*., 2011) can be used. In contrast to simple linear regression models, where the model parameters are fixed, LMMs include so called random effects that are assumed to follow a Gaussian distribution. In general, in LMMs the genetic marker to be tested and the covariates are modeled as fixed effects, while the genetic similarity between the individuals is modeled as a random effect. Let *c* be the number of fixed effects. Consider an LMM of the following form:


$$\begin{array}{l} y=X\beta +u+\varepsilon \end{array}$$

Here, $X\in R^{n\times c}$ is a matrix of fixed effects including a column of ones for the overall mean, the covariates and the SNP of interest. The vector $\beta \in R^{c}$ contains the corresponding effect sizes of the fixed effects, $u\in R^{n}$ are the random effects and the vector $\varepsilon \in R^{n}$ are the residual effects. It is assumed that ϵ follows a Gaussian distribution with zero mean and a covariance matrix $\sigma_{e}^{2}I\in R^{n\times n}$, where $\sigma_{e}^{2}$ is the residual variance component and $I\in R^{n\times n}$ is the identity matrix. We further assume that $u∼N\left(0,\sigma_{g}^{2}K\right)$ with genetic variance component $\sigma_{g}^{2}$ and kinship matrix $K\in R^{n\times n}$. It follows that y is also normally distributed with mean Xβ and covariance matrix $\sigma_{g}^{2}K+\sigma_{e}^{2}I$.

Similar to EMMAX (Kang *et al*., 2010), and FaST-LMM (Lippert *et al*., 2011), the variance components $\sigma_{g}^{2}$ and $\sigma_{e}^{2}$ are estimated once for the null model without genetic markers and used for the alternative model including the SNP of interest. (For more mathematical details on this procedure, see: Kang *et al*., 2010; Lippert *et al*., 2011; John *et al*., 2023b, Preprint.) An *F*-test is then performed to test the null hypothesis of no association against the alternative hypothesis that the marker does have an effect on the phenotype. If the resulting *P*-value is less than a predefined significance threshold α, the null hypothesis is rejected and we consider the statistical test to be significant. As mentioned in the introduction, an appropriate significance threshold is needed to correct for multiple tests performed and prevent thousands of false positive associations. For this, we propose to use an adjusted permutation-based significance threshold (Nicod *et al*., 2016; Ruzicka *et al*., 2019; Scott *et al*., 2021; John *et al*., 2022, 2023b, Preprint).

#### Permutation-based thresholds

To empirically estimate the FWER with permutation-based significance thresholds, one must consider two different permutation strategies, either permuting the phenotype vector or permuting the genetic marker (Fig. 1B, C). By permuting the phenotypic values, the correlation between them and the genotype is broken. Therefore, any signal remaining after the permutation is of non-genetic origin. However, when randomizing the phenotype, one ignores the underlying population structure and thus breaks the relationship between individuals. There are several approaches that try to avoid this, e.g. by computing the spectral decomposition first and permuting the transformed phenotype (Nicod *et al*., 2016; Ruzicka *et al.*, 2019; Scott *et al.*, 2021).

In general, when using a permutation-based approach, one aims to sample the true null distribution of the test statistics. For this, John *et al*. (2022) suggested using the *maxT* method proposed by (Westfall and Young, 1993). Here, the phenotype vector is permuted a certain number of times, and then the test statistics are computed for all permutations and genetic markers. Then, for each permutation, the maximum test statistic is taken and the corresponding minimum *P*-values are computed. The adjusted threshold is then defined as the αth percentile of the minimum *P*-values.

Although the resulting permutation-based significance threshold is better able to handle non-Gaussian distributions than the classical Bonferroni threshold (John *et al*., 2022), it should be treated with some caution. As mentioned above, when we randomize the phenotype, we ignore the underlying population structure and thus break the relationship between individuals. Therefore, John *et al*. (2023b, Preprint) suggested to permute not only the phenotype vector **y**, but also the rows and columns of the corresponding covariance matrix $\sigma_{g}^{2}K+\sigma_{e}^{2}I$ using the same permutation. This ensures that the estimates of our LMM parameters are still valid generalized least squares estimates. In fact, we have proven that this procedure is equivalent to permuting the fixed effects matrix X containing the covariates and the SNP of interest (John *et al*., 2023b, Preprint).

#### Efficient permutation-based GWAS

Regardless of the permutation strategy chosen, permutation-based GWAS are computationally expensive. Therefore, to efficiently compute univariate tests and permutation-based thresholds in batches, permGWAS2 has been developed (John *et al*., 2022, 2023b, Preprint). Instead of testing each SNP sequentially, permGWAS2 uses an efficient batch-wise reformulation of LMMs to compute several univariate tests simultaneously in 3D tensors. In addition, permGWAS2 supports multi-core and GPU architectures. This rigorously reduces the computational time required for a complete GWAS compared with classical GWAS tools such as EMMAX and FaST-LMM (Kang *et al*., 2010; Lippert *et al*., 2011).

To further accelerate permutation-based approaches, permGWAS2 simultaneously computes test statistics of multiple markers for different permutations using 4D tensors. By default, permGWAS2 permutes the fixed effects matrix X containing the SNP of interest and the covariates, to compute a permutation-based significance threshold, which is equivalent to permuting the phenotype and its covariance matrix as mentioned above. Since this approach requires additional computations for each batch of permuted SNPs, the computational costs are higher than for the simpler permutation strategy where only the phenotype is randomized in the beginning. To distinguish between the two permutation methods, in the following permGWAS2 refers to the default approach of permuting the phenotype and its covariance matrix, that is, permuting the SNP and covariates, and permGWAS2(y) refers to the simpler strategy, where only the phenotype y is permuted.

permGWAS2 is open source and can be downloaded from the following GitHub repository: https://github.com/grimmlab/permGWAS.

### Data

We analysed the performance of permutation-based GWAS on publicly available data from the model plant Arabidopsis well as simulated data.

#### Arabidopsis data

For our experiments, we used a fully imputed SNP matrix of 2029 Arabidopsis individuals with approximately 3M segregating markers as genotypic data (Arouisse *et al*., 2020). As phenotypic data we downloaded 536 publicly available traits from the public AraPheno database (Seren *et al*., 2016; Togninalli *et al*., 2020).

#### Synthetic data

A well-known limitation of LMMs is the assumption of Gaussian distributed residuals which is often violated in real-world data. To show the benefits of permutation-based approaches over classical methods such as the Bonferroni correction for non-normally distributed phenotypes, we simulated differently skewed traits. For our simulation experiments, we used 200 random individuals of the fully imputed Arabidopsis genotypes mentioned above. Based on these genomic data, we simulated artificial phenotypes with six different distributions, 100 simulations each. To create phenotypes with a certain amount of population structure, we used again an LMM, y=rmbetas+Cu+ε, as a base. For the polygenic background, we first computed the genetic similarity matrix via the relationship kernel and took the Cholesky decomposition $K=CC^{⊤}$. Then we multiplied C with a random vector $u\in R^{200}$ drawn from a Gaussian distribution with zero mean and a variance of 1. This ensures that the variance of these random effects is again equal to the kinship matrix K. To simulate phenotypes with a heritability of approximately 30%, we added a random noise vector $\varepsilon \in R^{200}$ such that the polygenic background contributed 30% of the phenotypic variance. For the random noise, we used either a zero mean Gaussian distribution or a gamma distribution with one of five shape parameters 4, 3, 2, 1 or 0.5. This results in six different simulation settings, with the phenotypes becoming more skewed as the shape parameter of the gamma distribution becomes smaller. Finally, as a fixed effect for each simulation, we added a different causal SNP, s, with a minor allele frequency greater than 5% and an effect size β to explain approximately 20% of the total phenotypic variance. Thus, for each artificial phenotype, we know the ground truth (i.e. the causal SNP).

### Experimental set-up

Unless noted otherwise, for each phenotype we ran permGWAS2 with 500 permutations. For our simulation experiments, we determined the number of true positives (TP) and false positives (FP) as a function of the threshold for each simulation. Since in Arabidopsis linkage disequilibrium decays on average within 10 kbp (Kim *et al*., 2007), we classified any significant SNP within a 10 kbp window around the causal marker as a TP. If a hit was detected outside this window, we classified it as an FP. To compare the performance of permGWAS2, permGWAS2(y), and Bonferroni, we computed the FDR of each simulation and threshold as FDR=FP/(TP+FP). In addition, we calculated the phenotype-wise FDR (pFDR) for each simulation setting and threshold. We defined a phenotype as a TP if a TP hit was found and as an FP if it had at least one FP association. Thus, a phenotype can be a TP and an FP at the same time. Then, the phenotype-wise FDR (pFDR) is given as the number of FPs per setting divided by the total number of positives.

## Results and discussion

To show the advantages of permutation-based GWAS approaches compared with classical models typically used for plant data such as EMMAX (Kang *et al*., 2010) and FaST-LMM (Loh *et al*., 2015), we performed new simulation and runtime comparison experiments. For this purpose, we first compared the performance of EMMAX and FaST-LMM with the two permutation-based LMM strategies described above in terms of computational runtime. We then evaluated the benefits of permutation-based significance thresholds over common approaches such as the Bonferroni threshold on simulated data. Afterward, we re-analysed all 536 publicly available Arabidopsis phenotypes with permGWAS2 to emphasize the advantages of permutation-based significance thresholds on real data.

### Computational runtime

**Fig. 2.**
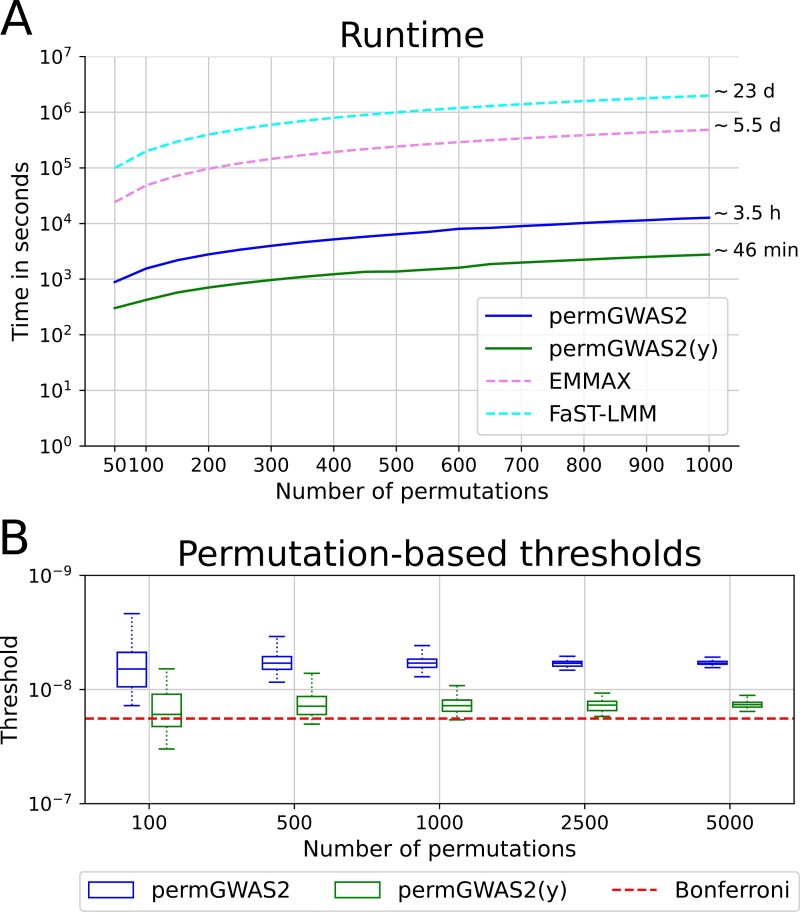
Comparison of runtime and permutation-based thresholds of permGWAS2 and permGWAS2(y) based on the number of permutations. (A) Computational time as a function of the number of permutations with fixed 1000 samples and 1 million single nucleotide polymorphisms (SNPs). Dashed lines for EMMAX and FaST-LMM are estimated based on the runtime for 1000 samples and 1 million markers times the number of permutations. (B) Permutation-based thresholds for different numbers of permutations on an inverted logarithmic axis. Thresholds were computed 50 times for the same phenotype. The static Bonferroni threshold is shown as a red dashed line.

To demonstrate the advantage of permGWAS2’s batch-wise formulation for computing permutations over state-of-the-art approaches, we first performed runtime experiments. For this purpose, we used 1000 samples of an arbitrary flowering time related phenotype of Arabidopsis and fixed the number of SNPs to 1 million of the corresponding genotype data (Arouisse *et al*., 2020). We ran permGWAS2 and permGWAS2(y) with between 50 and 1000 permutations on a single CPU and GPU. Since neither EMMAX nor FaST-LMM is intended to compute permutations, we ran them once and estimated the time by multiplying the actual runtime by the number of permutations. We repeated the experiments three times and took the mean computational time over all runs. The runtime as a function of the number of permutations is shown in Fig. 2A. With approximately 3.5 h and 46 min for 1000 permutations, both permGWAS2 versions clearly outperformed EMMAX and FaST-LMM, which both took several days. However, as expected, the permutation strategy of permGWAS2 was computationally more expensive than the simpler method of permGWAS2(y), where only the phenotype vector is permuted. Nevertheless, even with the more extensive permutation strategy of permGWAS2, the computation of permutation-based thresholds was feasible in practice. Even without using permutations, the batch-wise approach of permGWAS2 was much more efficient than its strictly sequential counterparts.

### Performance of permutation-based GWAS on simulated data

One of the main goals of this work is to analyse the advantages of permutation-based GWAS. To this end, we performed several experiments on simulated data. First, we give a recommendation on the number of simulations needed for a sufficiently accurate threshold. Then, we evaluate the performance of permGWAS2 compared with the classical Bonferroni significance threshold.

#### Number of permutations

When dealing with permutation-based methods, the first question that arises is how many permutations are needed to get sufficiently precise results. Obviously, the more permutations the better. However, even though permGWAS2 can compute many permutations in a short amount of time, the computational burden is still high for more than a few thousand permutations. Therefore, we need a recommendation on how many permutations are sufficient to get a good enough permutation-based threshold. To get an empirical estimate of the number of permutations needed, we performed the following experiments: for one phenotype with gamma distribution and shape parameter 1 from our simulations, we ran permGWAS2 and permGWAS2(y) 50 times with 100, 500, 1000, 2500, and 5000 permutations, and computed the permutation-based thresholds for a significance level of α=0.05.

The different thresholds as boxplots are summarized in Fig. 2B. As expected, the permutation-based thresholds stabilized with an increasing number of permutations for both permGWAS2 and permGWAS2(y). Surprisingly, regardless of the permutation method, 5000 permutations seemed to be sufficient to get a fairly stable threshold. For 100 permutations the values fluctuated a lot, which is especially problematic for permGWAS2(y) as the thresholds oscillate around the Bonferroni threshold. The range of values for 500 permutations was less than 1.9 × 10^−8^ for both settings. Considering also the previous runtime experiments, where permGWAS2 took about 107 min for 500 permutations and permGWAS2(y) took only 23 min, 500 permutations seemed to be a good compromise. So we used 500 permutations for the following experiments.

#### Comparison of permutation-based thresholds

**Fig. 3.**
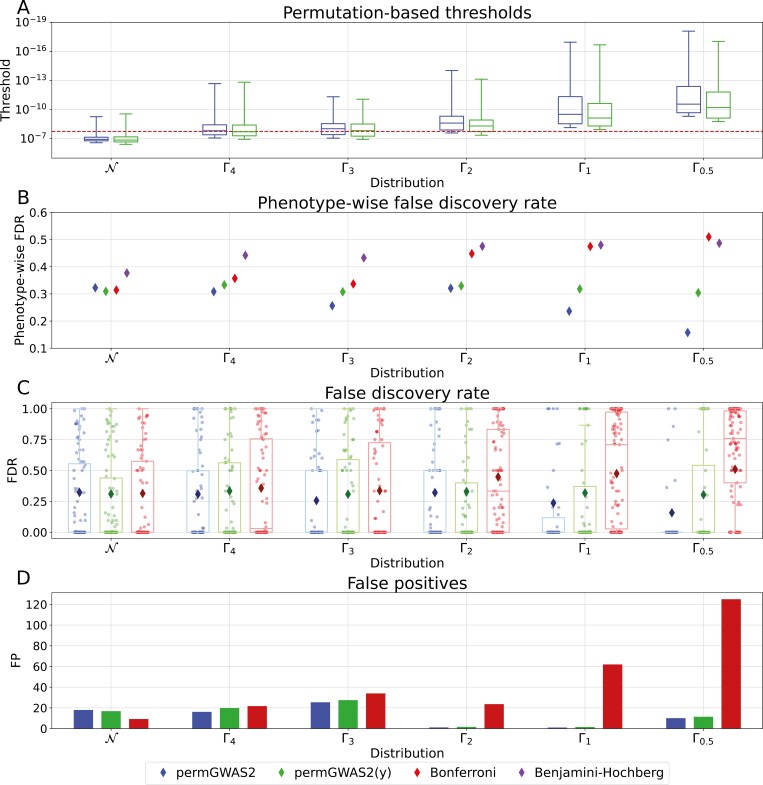
Comparison of permGWAS2 and permGWAS2(y) with Bonferroni threshold on simulated data with normally (N) or gamma-distributed noise with different shape parameters (Γ_4_, …, Γ_0.5_). (A) Permutation-based thresholds over 100 simulations as box plots for each distribution. Red dashed line shows the static Bonferroni threshold. (B) Phenotype-wise false discovery rate (FDR) for permutation-based thresholds, Bonferroni, and Benjamini–Hochberg significance threshold. (C) FDR for each simulation as strip plots. Box plots visualize the overall distribution and diamonds represent the corresponding phenotype-wise FDR. (D) Average number of false positives over 100 simulations per distribution.

Based on the previous results, we ran permGWAS2 and permGWAS2(y) with 500 permutations for all 600 simulated phenotypes. Figure 3A shows the permutation-based thresholds on an inverted logarithmic axis as a function of the distribution of the simulated phenotype. Compared with the static Bonferroni threshold at 1.79 × 10^−8^, both permutation-based thresholds were generally less conservative. From left to right, as the phenotypes became more skewed, the permutation-based thresholds became more stringent, with permGWAS2 being even stricter than permGWAS2(y).

#### Phenotype-wise false discovery rate

The pFDR for permGWAS2, permGWAS2(y), and Bonferroni is summarized in Fig. 3B. For permGWAS2(y) the pFDR seemed to be stable between 0.3 and 0.35 for all distributions. In contrast, the pFDR decreased visibly for more skewed phenotypes with permGWAS2. The Bonferroni threshold showed a similar pFDR to permutation-based thresholds when the phenotypes were normally distributed. However, for skewed phenotypes, the pFDR increased to more than 0.5, meaning that we found as many FPs as TPs. Therefore, especially permGWAS2 seems to be better able to control false positive associations compared with the classical Bonferroni threshold. We also compared the three thresholds mentioned above with the FDR-based Benjamini–Hochberg method (Benjamini and Hochberg, 1995), which, unlike FWER-based methods, tries to control the FDR. Notably, the pFDR with Benjamini–Hochberg was larger than with the other methods for Gaussian distributions and similar to Bonferroni for the most skewed cases. These results are not surprising, as the Benjamini–Hochberg method generally provides a less stringent control of FPs than FWER-based methods. The Benjamini–Hochberg procedure computes an adjusted significance threshold based on the ranked *P*-values. However, in the skewed case, the *P*-values are incorrect due to model misspecifications, as the assumption of a Gaussian distribution is violated. Hence, the Benjamini–Hochberg approach is not applicable in this situation.

#### Simulation-wise false discovery rate

We compare the FDR of all individual simulations for permGWAS2, permGWAS2(y), and Bonferroni (Fig. 3C). Here, each dot represents a simulation with at least one hit—TP or FP—and a boxplot visualizes the overall distribution of the FDR. Additionally, a diamond represents the pFDR for comparison.

As expected, with the Bonferroni threshold we generally got more hits the more skewed the phenotypes were, implying that Bonferroni is rather conservative for Gaussian distributions and not stringent enough for skewed ones. Both permutation strategies allow fewer hits for gamma distributions with smaller shape parameters. However, for all distributions the permutation-based thresholds achieve FDRs of 0 in more than 50% of the cases with hits, meaning that we only found TPs for these simulations. In the most extreme case—the gamma distribution with shape 0.5—permGWAS2 yielded an FDR of 0 for 82% of the phenotypes with hits compared with 16% for Bonferroni and 65% for permGWAS2(y). Furthermore, the individual FDRs with Bonferroni were getting closer to 1 for the more skewed simulations.

Finally, the average number of FPs per simulation setting is shown in Fig. 3D. Clearly, with Bonferroni, the number of FPs increases as the phenotypes become more skewed, with an average of more than 120 FPs in the most extreme case. On the other hand, with permutation-based thresholds the average number of FPs was less than 30 for all simulation settings.

In summary, the simulation experiments demonstrate that permutation-based methods are better at controlling FPs for skewed phenotypes than the classical Bonferroni threshold or FDR-based methods such as Benjamini–Hochberg.

### The effect of permutation-based significance thresholds on real Arabidopsis data

To illustrate the usefulness of a permutation-based significance threshold on real data, we re-analysed all 536 publicly available Arabidopsis phenotypes from the public AraPheno database (Seren *et al*., 2016; Togninalli *et al*., 2020) using permGWAS2 with 500 permutations each. A summary of our results, including the Bonferroni and permutation-based threshold and respective number of significant associations for each phenotype, is shown in Supplementary Table S1.

As described in the previous section, the permutation-based threshold is not static but depends on the phenotypic distribution. We expected that for phenotypes that are normally or nearly normally distributed, a permutation-based threshold may be less stringent than a static threshold that only considers the number of tests performed, such as the Bonferroni significance threshold. In contrast, for non-normal, skewed, or categorical phenotypes, the permutation-based threshold should reflect the model misspecification introduced by the distribution and should be more stringent. In fact, for 198 of the 536 phenotypes that we re-analysed, the permutation-based threshold was less stringent (i.e. higher) than the static Bonferroni threshold, while for 338 phenotypes we observed a more stringent threshold.

Statistically, even more important than the actual phenotypic distribution is the distribution of the residuals, which is assumed to be Gaussian in linear models. If the residuals are not normally distributed, the test statistics can easily be inflated and the null hypothesis can spuriously be rejected. Therefore, we wanted to test whether this violation of the model assumption, caused by non-normal phenotypic distributions, affects the stringency of the permutation-based threshold in real data.

#### Thresholds for non-normal distributions

**Fig. 4.**
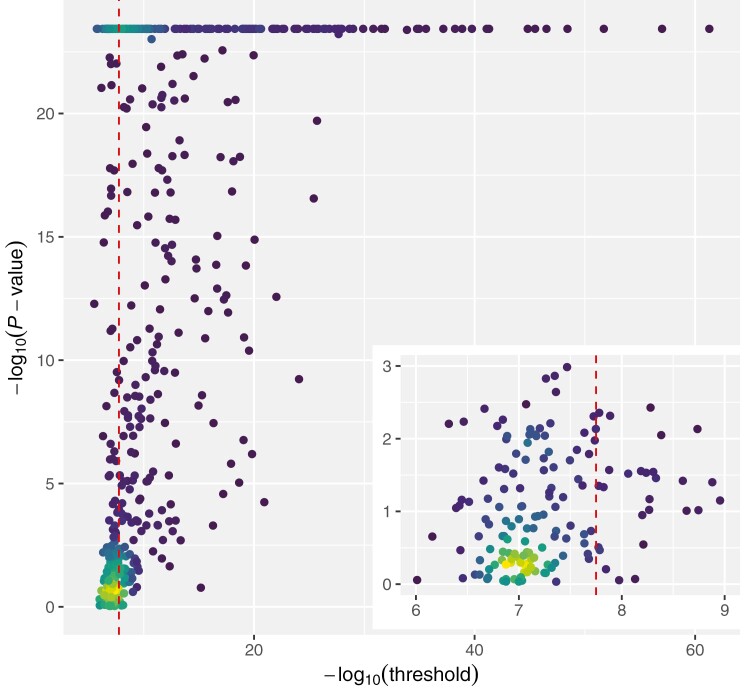
Comparison of the permutation-based 5% significance threshold and the test statistic from the Anderson–Darling test (AD). The −log_10_(threshold) of the permutation-based significance threshold is plotted against the −log_10_(*P*-value) from the Anderson–Darling test on the phenotypic distribution. The red, vertical dashed line reports the Bonferroni threshold for testing 2.8 million SNPs.

A commonly used statistical method to test for a normal distribution of the residuals is the Anderson–Darling test (Anderson and Darling, 1954). The Anderson–Darling test is well suited for assessing the normality of residuals due to its increased sensitivity. Unlike some other tests, it gives more weight to deviations in the tails, making it effective at detecting subtle deviations from normality. This sensitivity is critical when evaluating the assumptions of regression models, where outliers or non-normality in the residuals can affect the validity of statistical inferences. The Anderson–Darling test’s emphasis on extreme values enhances its ability to identify deviations from normality in the tails, making it a valuable tool for robust residual analysis in statistical modeling (Loy and Hofmann, 2015). Therefore, we performed an Anderson–Darling test on all 536 phenotypes and plotted the respective *P*-values against the calculated permutation-based threshold in a 2D-density plot (Fig. 4). The permutation-based threshold was less stringent for phenotypes that are closer to a normal distribution (Fig. 4, inset, i.e. where the *P*-values of the Anderson–Darling test are larger than 0.01), while it became increasingly stringent as the *P*-values of the Anderson–Darling test decreased. Thus, we can see that the permutations provide a threshold that depends on the phenotypic distribution and tends to decrease as model assumptions are more violated. But what is the real effect of this threshold on the number of significant associations reported? If we now compare the number of reported significant associations using the permutation-based threshold with the number of reported significant associations using the Bonferroni threshold, we observe differences depending on the phenotype, or more precisely, the phenotypic distribution.

#### Distinct categories of results

Because we need to compare several different traits, it is difficult to make assumptions about trait architecture in general. Some traits may be Mendelian, others may be highly polygenic. For the latter, it is not trivial to estimate the number of true hits one would expect in a best-case scenario. Using simulations (Korte and Farlow, 2013) we roughly know the power of GWAS to identify effects of a given size given the sample size. If we apply this to the traits analysed, we know that in a best case scenario we can only detect a maximum of 10 independent associations. This estimate is potentially far too conservative, but gives a good indication of how to summarize across a large number of different traits. Therefore, we considered traits with up to 10 independent associations as having a reasonable number of associations (i.e. 10 different regions with significant associations that are more than 10 kbp apart), since linkage disequilibrium decays in Arabidopsis within 10 kbp (Kim *et al*., 2007). Based on this assumption, and without discussing each individual phenotype, we can roughly divide the phenotypes into seven different categories (Table 1). These are the following.

**Table 1.** Results of comparing permutation-based thresholds with Bonferroni thresholds: in the re-analyses of 536 available Arabidopsis phenotypes, seven distinct scenarios are observed

Scenario	Cases
Significant hits only with Bonferroni	168
Significant hits only with permutations	19
Reasonable amount with both methods	51
Inflation with permutations, reasonable with Bonferroni	7
Inflation with Bonferroni, reasonable with permutations	51
Visible inflation with both thresholds	38
No hits independent of the threshold	202

The first column describes the scenario, where we assume that up to 10 independent significant hits are reasonable. The last column shows the total number of phenotypes in the respective category.

**Fig. 5.**
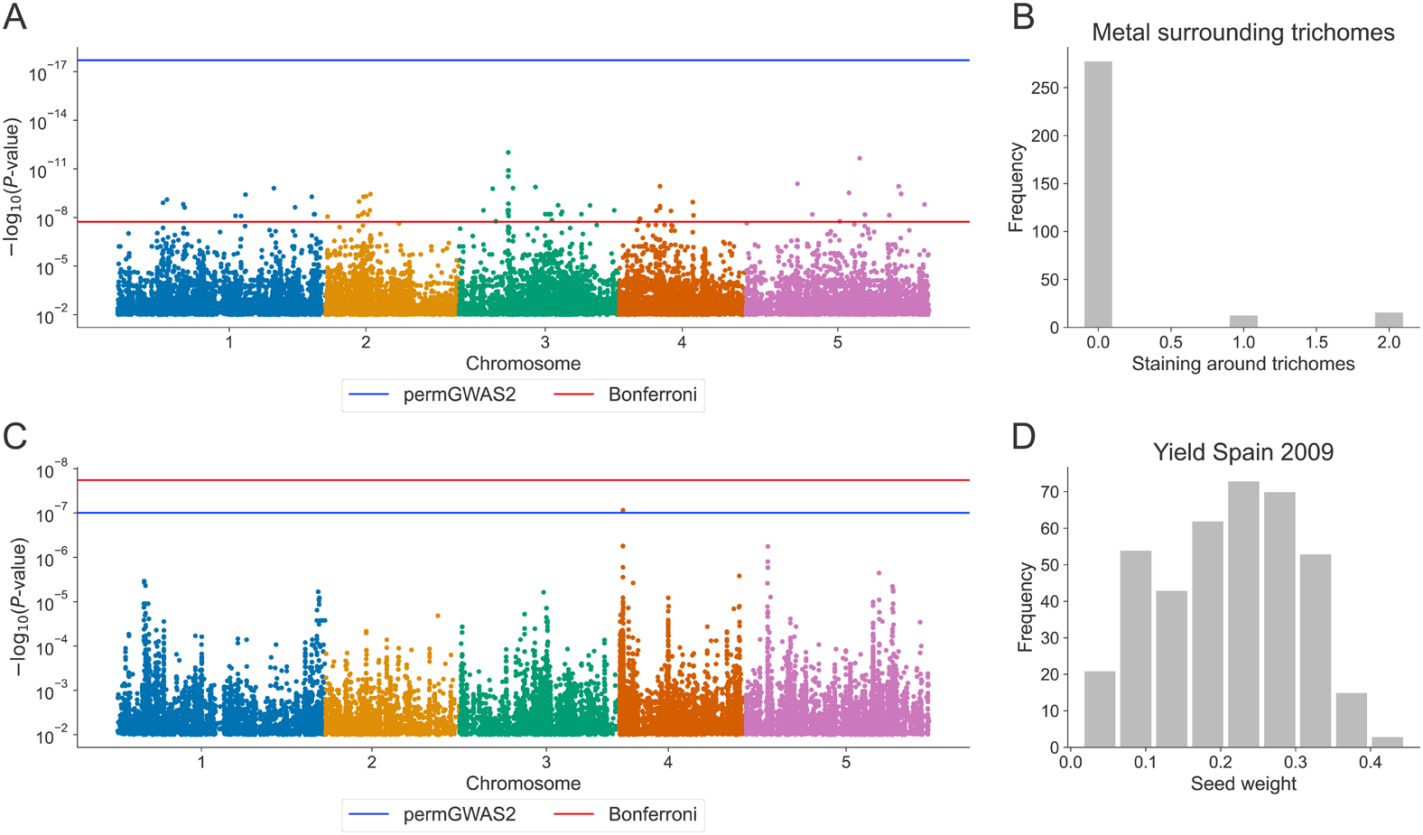
permGWAS analyses of two different Arabidopsis traits. (A) Manhattan plot of the phenotype ‘Metal surrounding trichomes’. (B) Phenotypic distribution of the phenotype ‘Metal surrounding trichomes’. (C) Manhattan plot of the phenotype ‘Yield in Spain 2009’. (D) Phenotypic distribution of the phenotype ‘Yield in Spain 2009’.

(i) Significant hits are detected after applying a Bonferroni threshold, but not after applying the permutation-based threshold. This category, which contains 168 phenotypes, highlights that for many non-normally distributed phenotypes the Bonferroni threshold is not stringent enough and associations that are below this threshold will contain many false positive associations, due to model misspecification. An example of a Manhattan plot of such a phenotype is shown in Fig. 5A with corresponding phenotypic distribution in Fig. 5B. This Manhattan plot is representative of many phenotypes in this category that show clearly inflated results if the Bonferroni threshold is used.
(ii) Significant hits are detected after applying a permutation-based threshold, but not after applying the Bonferroni threshold. There are 19 phenotypes in this category. Here, the Bonferroni threshold was too stringent, and a permutation-based threshold allows for the detection of novel associations. An example of a Manhattan plot for a phenotype with a new association is shown in Fig. 5C and will be discussed in more detail later. Note that these phenotypes tend to be normally distributed (see Fig. 5D), in contrast to the clearly skewed distribution of phenotypes belonging to category (i) (Fig. 5B).
(iii) A reasonable number of significant associations (up to 10 independent loci) are detected with both thresholds. There are 51 examples of this scenario in the Arabidopsis data.
(iv) Even after using a permutation-based threshold, inflated results are observed, whereas no inflation is visible with Bonferroni. For seven phenotypes, the permutation-based threshold was unable to account for model misspecification. Permutations are not a panacea, although they generally do a good job.
(v) The use of a Bonferroni threshold produces inflated results, whereas a more stringent permutation-based threshold—in this case—clearly reduces the number of associations to a reasonable level (i.e. at most 10 independent ones). Here, there is a high probability that these associations are indeed true, and it would be worthwhile to test them or perform follow-up experiments. Fifty-one phenotypes fell into this category.
(vi) For 38 phenotypes both the Bonferroni threshold and the permutation-based threshold produce visibly inflated results.
(vii) For 202 phenotypes, we could not detect a significant association. The main reasons could be a lack of statistical power due to a small sample size or small effect loci. In these cases, there may still be false negatives, but a larger sample size would be needed to identify them. In addition, if a trait is not heritable, e.g. the phenotypic measurements were not robust, we probably do not expect or want GWAS to detect associations.

In summary, the re-analyses of the Arabidopsis data revealed scenarios where the use of a permutation-based threshold was beneficial in reducing false positives as well as in identifying novel associations. Anecdotally, in the example shown in Fig. 5C, the novel association found indeed makes biological sense. The significant association is located on chromosome 4 at position 552 431 in an intron of the gene AT4G01330. This gene belongs to the protein kinase superfamily, is highly expressed in flowers, and is associated with the GO terms growth and response to light intensity. It has even been identified as a hub gene in the Arabidopsis light stress signaling network (Huang *et al*., 2019). The corresponding phenotype scored the yield as dry seed weight of plants grown in simulated seasons mimicking a high-light environment in Spain (Li *et al*., 2010; Li, 2016).

### Conclusion

In this review, we discussed two permutation strategies, population-aware and population-unaware permutations, as alternatives to the commonly used Bonferroni correction to account for multiple hypothesis correction in genome-wide association studies. The first strategy permutes the phenotype vector as well as the rows and columns of the corresponding covariance matrix, which is equivalent to permuting the fixed effects matrix including the SNP of interest and the covariates. The second strategy permutes only the phenotype vector, which breaks the population structure between samples. For this study, we performed new simulations to compare the benefit of permutation-based significance thresholds to the commonly used and static Bonferroni threshold. Our simulations for phenotypes with differently skewed distributions showed that permutation-based methods are better at controlling the false discovery rate than the classical Bonferroni correction or FDR-based methods such as Benjamini–Hochberg. The population-aware strategy permGWAS2 yielded even smaller false discovery rate values than permGWAS2(y) at the cost of a (slightly) increased runtime. In general, permutation-based methods lead to fewer hits compared with classical Bonferroni correction for skewed phenotypes, but with an increased probability that a significant association is indeed a true positive. As false positives are a bigger concern than false negatives for most studies—especially in plant biology, where functional follow-up studies are possible but time consuming—the reduced power is an acceptable price to pay. Furthermore, we showed that permutation-based GWAS can be efficiently computed in a batch-wise approach using 4D tensors, and even outperforms state-of-the-art GWAS approaches in terms of runtime when no permutations are used. We also empirically estimated the number of permutations required to obtain a fairly stable significance threshold. Based on our results, we recommend performing at least 500 permutations if computational resources are limited. In summary, we have shown in this review that permutation-based significance thresholds provide an alternative strategy for correcting for multiple hypotheses that results in a lower false discovery rate compared with the classical Bonferroni correction for skewed phenotypic distributions, and can be readily implemented with available tools. In this context, it is noteworthy that skewed, non-normal distributions are common for many plant phenotypes. In the Arabidopsis data we analysed, over 80% (435/536) of the phenotypes were non-normally distributed.

## Supplementary data

The following supplementary data are available at *JXB* online.

Table S1. Summary of permutation-based GWAS results of 536 Arabidopsis phenotypes downloaded from AraPheno database.

erae280_suppl_Supplementary_Table_S1

## Data Availability

The genotype data are available at: https://figshare.com/projects/Imputation_of_3_million_SNPs_in_the_Arabidopsis_regional_mapping_population/72887. The phenotype data are publicly available at: https://arapheno.1001genomes.org. permGWAS is publicly available in GitHub: https://github.com/grimmlab/permGWAS.
